# From microscopes to maps: enabling a new era of open and reproducible data sharing in intravital imaging

**DOI:** 10.1038/s44318-025-00630-x

**Published:** 2025-11-20

**Authors:** Moritz Peiseler

**Affiliations:** Department of Hepatology and Gastroenterology, Charité - Universitätsmedizin Berlin, Campus Virchow Klinikum and Campus Charité Mitte, Berlin, Germany

**Keywords:** Computational Biology, Immunology, Methods & Resources

## Abstract

New research in *The EMBO Journal* shows how the publicly accessible database Immunemap can facilitate the study of immune cell motility.

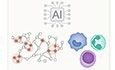

## Teaser

Understanding the motility of immune cells and their interactions with their environment in response to different stimuli and in various organs has been a key objective in immunology. In this issue of *The EMBO Journal*, Pizzagalli and colleagues present proof-of-principle analyses of immune cell motility using data from the curated and publicly-accessible intravital microscopy database “Immunemap”, which includes imaging data from 20 leading laboratories.

The immune system has a central role in protecting the organism against potentially detrimental infections. In addition to microbial invasions, tissue injury or toxic compounds can also trigger an inflammatory response, resulting in sterile inflammation. In recent years, the immune system is increasingly viewed as a diverse and complex system that transcends defense mechanisms actively participating in many physiological processes, such as metabolism, thermogenesis, circulation, or function of the nervous system (Medzhitov, 2021; Nahrendorf et al, 2025). To fulfill these diverse functions, the immune system employs specialized tissue-resident immune cells in organs, such as tissue-resident macrophages, innate lymphoid cells, or tissue-resident T cells. These cells are imprinted by the tissue environment and deeply integrated into organ function. As such, microglia, the tissue-resident macrophages of the central nervous system, exhibit a unique gene signature compared to other tissue macrophages despite originating from a common progenitor cell during fetal development (Lavin et al, 2015). The tissue-resident arm of immunity is complemented by migratory leukocytes, which travel the body as patrolling cells through blood and lymph vessels to act as an integrative system connecting organs throughout the body. An inflammatory response to infectious agents or danger signals leads to a massive influx of migratory leukocytes from the circulation and other compartments of the body. Understanding of immune cell motility patterns and interactions with their environment has therefore been a key goal in immunology.

**Figure 1:**
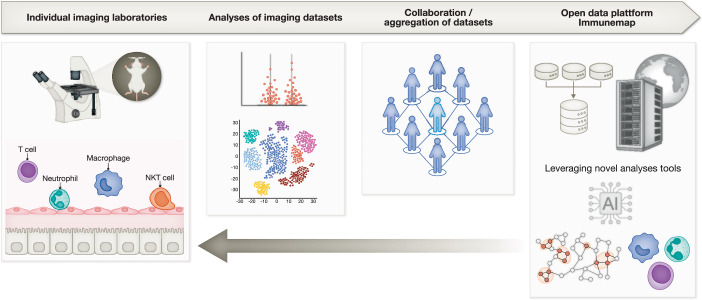
Immunemap overview. Intravital imaging enables the dynamic assessment of immune cell motility in an unperturbed tissue environment. The combination of imaging data from 20 world-leading laboratories allowed the creation of Immunemap, an open-data platform harboring curated datasets of single-track analyses in an accessible web-based format. Immunemap adheres to the FAIR principles and fosters cross-disciplinary collaboration.

Intravital microscopy is a method, ideally suited to capture all unique properties of immune cells, such as migration patterns, crosstalk, and function, while studying cells in their unperturbed tissue environment (Wang et al, 2024). In contrast, other approaches such as traditional histology or modern single-cell or spatial transcriptomics, while providing great granularity at the individual cell level, capture a single timepoint, thus overlooking the dynamic and temporal aspect of the inflammatory response. Two cells that appear closely colocalized in a static image may either be passing by each other at random, or alternatively engaged in prolonged interactions such as immune synapse formation (Mempel et al, 2004), a key difference that can only be resolved with dynamic intravital microscopy. The field of intravital microscopy has come a long way since its beginning over 100 years ago, with technical advancements such as confocal and multiphoton microscopy, imaging windows, and sophisticated genetic reporter systems (Wang et al, 2024). Spectral imaging now offers true multiplex intravital imaging, resulting in massive datasets that require customized and sophisticated computational solutions to perform image analysis. With machine learning and artificial intelligence as the next technological revolution on the horizon, and already being increasingly implemented in the field (Peiseler et al, 2023), data storage, handling, and access will be a critical issue to be addressed by the community. This requires robust and scalable data management frameworks and a collaborative spirit from individual laboratories. At the moment, only a fraction of selected data is made available to the public, and most raw imaging datasets are stored on private hard drives or servers, restricting access to the broader scientific community. This limits the reuse, reproducibility, standardization, and large-scale analyses of intravital microscopy data. The omics field has led the revolution towards making data publicly available for reuse and discovery thus democratizing data and fostering cross-disciplinary collaboration.

To address this important issue, a consortium of leading imaging experts has generated “Immunemap” (https://www.immunemap.org), an open-data platform of intravital microscopy data, delivering a curated and publicly accessible database for the community. Immunemap has a cloud-based architecture and offers easy access through a browser-based web application. An international consortium of 20 laboratories with distinct intravital microscopy expertise has collaborated and provided imaging data to be leveraged and stored in Immunemap. Importantly, the platform hosts imaging data, metadata (i.e., information about the microscope, settings, pixels, acquisition settings, stainings, channels, mouse models), experimental details, and single-cell tracks. Immunemap also supports advanced computational analyses by providing application programming interfaces (APIs) to allow seamless integration with common analysis tools, such as R, Python, and Matlab (Fig. 1).

In this issue of *The EMBO Journal*, Pizzagalli and colleagues leverage over 400 intravital videos—over 58,000 curated single-cell tracks and more than 1,049,000 cell-centroid annotations—stored in Immunemap to investigate migratory behaviors of different immune cells across multiple tissues and conditions. The findings presented in the manuscript offer a glimpse of the potential that Immunemap offers to researchers. An analysis of immune cell motility revealed CD8^+^ T cells as cells with the highest median speed, while dendritic cells were the least motile. Furthermore, while tracking analysis is often restricted to 2D video sequences, comparative assessment of migration patterns in 2D and 3D revealed that dimensional reduction to 2D can introduce bias as well as particularly underestimated cell displacement and mean speed compared to 3D assessment (Pizzagalli et al, 2025). Extending their targeted motility analyses, the authors used unsupervised machine learning of almost 15,000 tracks relying on an algorithm based on density peaks and network theory (Pizzagalli et al, 2019) to identify ten distinct motility patterns. Subsequent correlation of motility patterns with cell identities revealed broad properties of major immune cells—in some cases overlapping—and particularly of so far understudied immune cells such as eosinophils and γδ T cells. Importantly, the analyses of motility patterns were done at the steady state, during different inflammatory conditions and in different organs, confirming previous studies that had reported distinct motility patterns of immune cells based on the organ site of recruitment (McDonald et al, 2008). Studies based on intravital microscopy are often affected by the technical properties of the microscope and the experimental settings. By leveraging the data collected in Immunemap, the authors could demonstrate that the temporal sampling rate of time-lapse sequences had a striking impact on the measured cell velocity, with longer intervals seeming to underestimate true velocity.

Immunemap offers the potential to reshape the future of intravital imaging and the broader field of immunology in multiple ways. Indeed, Open Data initiatives guided by the FAIR principles (Findability, Accessibility, Interoperability, and Reusability) are becoming important in sharing scientific data (Wilkinson et al, 2016). Particularly with regard to rare cell types, in the single-cell transcriptomics field it was shown that combining multiple datasets can result in the identification of rare cell types otherwise overlooked in individual datasets (Xu et al, 2024). Using Immunemap, one can assume that the collected data will allow to establish characteristics of immune cells in different conditions by combining multiple datasets, thus identifying subsets of the major immune cells hitherto unknown. Currently, most intravital microscopy experiments are performed in the context of hypothesis-driven research. A large and growing dataset will allow researchers to leverage AI and machine learning to perform unbiased analyses, resulting in insights not predicted or expected. As data become accessible for reuse, this also supports the concept of 3R by reducing the number of animals needed for an experiment or replacing the planned experiment with reuse of existing data. Another beneficial aspect might lie in improved standardization and reproducibility of intravital microscopy. Currently, most imaging labs have their respective models, labeling strategies and experimental approaches. Harmonizing data sharing could help the community to establish standards resulting in more comparable datasets, in turn allowing researchers to rely on larger datasets for complex analyses. Uploading data to Immunemap will contribute to their standardization by requiring curation, quality scoring, and the description of experimental metadata. Beyond the core intravital microscopy community, Immunemap also provides access to dynamic immune cell motility data to immunologists that do not use intravital microscopy as their core technique. Given the cost of a state-of-the-art microscope, it may be in many cases sufficient to link transcriptomic data with motility patterns available through Immunemap, rather than performing de novo intravital microscopy experiments. This should enhance cross-disciplinary collaboration. This resource will be available in a growing repository of high-quality videos and expert-curated motility patterns with additional tools for further analysis. As a public repository, Immunemap will provide an opportunity for computational training and education.

While a fantastic resource, Immunemap still faces challenges. The 20 participating laboratories likely contributed highly selected videos, which may have skewed the presented quality score, the tracking analyses, and conclusions regarding cell migration in different organs. In addition, similar to transcriptomic repositories, the reuse of published data is not free of the bias of data heterogeneity, context loss, overinterpretation of selected data, or inconsistent metadata. Data generated by the intravital microscopy community are often unique to the group that produced them given that the microscopy modalities (two-photon, confocal, spinning disk, upright, inverted), labeling strategies (fluorescent reporters, antibodies, adoptive transfer of labeled cells), and surgical methods (imaging preparations, mode of anesthesia etc.) vary between laboratories (Wang et al, 2024). Combining multiple datasets will thus inherently suffer from data heterogeneity. Unfortunately, with the present set of videos, Immunemap does not contain sufficient datasets of identical combinations of cell types, organs, and models, which limits the direct comparison under matched conditions. However, with a growing repository, researchers might soon be able to compare the same cell type in the same organ, obtained with different microscopes in different laboratories.

Immunemap is a fantastic resource for the imaging community and beyond, and should grow in value as more groups contribute more datasets. The true value of Immunemap lies within the open-data platform infrastructure with curated datasets, comprehensive metadata, and computational tools. The opportunities of such a platform will enable new and data-driven research centered around the dynamic behavior of immune cells in a spatiotemporal context. In the current issue of *The EMBO Journal*, Pizzagalli et al provide interesting first findings, leveraging Immunemap to identify novel and tissue-specific migratory patterns of immune cells. Combining the dynamic in vivo imaging datasets with single-cell and spatial transcriptomic datasets will enable researchers to enter a new era of decoding biological phenomena at a tissue level and in a dynamic fashion.
